# Supplementary material for: Comparative performance evaluation of Wako β-glucan test and Fungitell assay for the diagnosis of invasive fungal diseases
Source: PLoS One. 2020 Jul 29;15(7):e0236095. doi: 10.1371/journal.pone.0236095 (PMC7390339; doi:10.1371/journal.pone.0236095)
Supplement: Attachment — Submitted filename: PONE-D-19-33241R1 Response to Reviewers.doc [file pone.0236095.s006.doc]

PONE-D-19-33241R1

Reviewer #4: Most of my comments have been addressed. However, I disagree with the reply to comment 10 regarding statistical analysis. Overall, Reviewer #3's comments are largely the same as mine. We both state that you cannot make any proclamation about superiority without doing due analysis. However, this does not mean this is not possible, or indeed, that this can be waived!

The fact that this is a head-to-head analysis means a pairwise comparison is indicated, such as McNemar's test. Using this test, you can confidently state for example, that the sensitivity of FA > 80 is significantly greater than the sensitivity of GT > 7 (92.5% vs 80%, p=0.025). Most statistical packages have tools specifically for comparing two diagnostic tests.

**Answer: We are delighted in noticing that we were able to satisfy most of the reviewer’s comments. Regarding the unsatisfied remaining comment raised by the reviewer (also in line with the reviewer #3), we treasured the suggestion of the reviewer. Thus, we used the McNemar's test to assess statistically the differences in performance parameters between FA and GT. Accordingly, we added five sentences, one of which in the Materials & Methods section (Statistical Analysis paragraph) and other four in the Results section (Performances of the FA and the GT). See the revised version of the manuscript (PONE-D-19-33241R1 with Track Changes).**
